# Identification of the Influence of IPA Rinsing Times on Surface Roughness of SLA-Printed Parts Made of Different Materials

**DOI:** 10.3390/ma18092082

**Published:** 2025-05-01

**Authors:** Wiktor Harmatys, Adam Gąska, Piotr Gąska, Maciej Gruza

**Affiliations:** 1 Cracow University of Technology, Faculty of Mechanical Engineering, Laboratory of Coordinate Metrology, 24 Warszawska Street, 31-155 Cracow, Poland; wiktor.harmatys@pk.edu.pl (W.H.); maciej.gruza@pk.edu.pl (M.G.); 2 AGH University, Faculty of Mechanical Engineering and Robotics, Department of Manufacturing Systems, al. Mickiewicza 30, 30-059 Krakow, Poland; gaska@agh.edu.pl

**Keywords:** sterolitography, roughness, post-processing

## Abstract

This study investigates the influence of isopropyl alcohol (IPA) washing time on the surface roughness of stereolithography (SLA)-printed parts fabricated using the Formlabs Form 3B+ printer. Three photopolymer resins provided by the manufacturer were evaluated: Gray, Tough 2000, and Rigid 10K. Samples were printed in standardized geometries and post-processed under controlled conditions, with IPA washing times ranging from 6 to 30 min, followed by UV post-curing. The surface roughness parameters (Ra, Rz, Rt, and RSm) were measured using a Taylor Hobson Form Talysurf i-Series profilometer under metrologically controlled conditions. The results revealed a clear correlation between increased IPA exposure time and improved surface finish, though the magnitude and monotonicity of this effect were material dependent. Rigid 10K exhibited the most consistent reduction in roughness with longer washing, while Tough 2000 showed substantial improvement with extended durations but also demonstrated temporary surface degradation at intermediate wash times. Gray resin achieved near-optimal roughness after moderate rinsing, with orientation-dependent differences observed. The findings indicate that the careful optimization of washing duration can significantly enhance the surface quality in SLA prints, potentially eliminating the need for secondary finishing processes. The implications are relevant to both industrial and medical applications where dimensional fidelity and surface smoothness are critical. Recommendations for optimal washing durations are proposed for each material, and directions for further research are outlined.

## 1. Introduction

In recent years, additive manufacturing (AM), particularly stereolithography (SLA), has revolutionized the fabrication of complex geometries and custom-designed components, with applications spanning across various sectors such as biomedical engineering [1], aerospace [2], or precision tooling [3]. SLA, a highly precise form of vat photopolymerization, utilizes photopolymeric resins cured by ultraviolet (UV) light to produce components with a superior surface resolution compared to other AM technologies [4,5]. However, despite the advancements in SLA, achieving the optimal surface finish remains a critical challenge due to the layer-by-layer construction of parts, leading to anisotropic surface textures and varying degrees of roughness depending on the orientation of the build [6]. SLA is a method that usually needs post-process operations in order to obtain the assumed goals and print characteristics. A number of research papers state that post-processing techniques such as UV curing [7] are essential for advancing the technology toward more demanding applications [8].

The surface roughness of printed components is one of the most important factors that influences their mechanical properties, functionality, and overall esthetic appeal [9]. In many industrial applications, surface smoothness is crucial not only for mechanical performance, such as reducing friction and wear in moving parts, but also for ensuring the compatibility of parts in assembly processes or maintaining tight tolerances in high-precision applications. The surface texture of SLA-manufactured elements depends on many factors related to each stage of the printing process, including process planning, its execution, and post-process activities. In [10], Ligon et al. discuss the importance of selecting polymer materials for 3D printing, including SLA. The authors state that different photopolymer resins are characterized by different mechanical and chemical properties, which affect the final surface roughness after printing. Additionally, the article emphasizes that the optimization of processing conditions, such as UV exposure time, is crucial to obtain a surface with low roughness. In [11], Reeves and Cobb discussed the influence of print orientation on the surface characteristics, indicating that the use of surface orientations close to perpendicular in relation to the device’s XY plane (assuming that the vertical direction is represented by the Z axis) can significantly reduce surface roughness, as the effects of stair stepping become less dominant. The authors also presented how surface roughness can be improved by modifying the fabrication process in order to obtain the so-called Meniscus Smoothing effect, which can be beneficial for surface orientations close to the direction parallel to the XY plane of the device. In [12], Khodaii and Rahimi also tested the influence of surface orientation on the printed element quality, stating that the surface roughness for horizontally oriented surfaces is similar to that obtained for surfaces arranged vertically. However, for surface orientations different from horizontal, the Ra parameter value increases rapidly and continues to grow to an approximately 30° inclination angle of the surface in relation to the vertical build orientation. After this, the roughness value becomes smaller, achieving its smallest value at vertical orientation.

Another factor that may have an impact on the surface quality is the layer thickness utilized during the fabrication process. In [13], Mukhangaliyeva et al. investigated this factor’s influence, as well as that of several others on the results of the manufacturing process of hip implants. The authors state that layer thickness was second factor to have the biggest impact on the surface roughness after the build angle, and that by decreasing it, it is possible to obtain a smoother surface. Also, in [14], Zhao et al. showed that a reduced layer thickness contributed to the better quality of the surface texture. Three layer thickness values were used in their studies for the fabrication of standardized specimens. The measured Ra value for specimens characterized by an 0.08 mm layer thickness was reduced by 10%, comparing one obtained element with a 0.12 mm layer thickness. In the same article, the authors compared the roughness of elements that were post-cured with UV, utilizing different curing methods and objects made without post-curing. The results of the presented experiments suggest that UV curing tends to increase the surface roughness. Park et al. [15] discuss the effect of UV irradiation on the mechanical properties and surface roughness of SLA prints. The authors conducted studies on polymer systems with different monomers, finding that a prolonged exposure time combined with high temperature can lead to surface cracking or increased roughness. This study shows that although UV curing increases the hardness and mechanical stability of printed parts, the excessive use of it can cause surface degradation problems.

A different approach to studying SLA surfaces was presented by Peng et al. [16], who focused on post-processing processes such as grinding, chemical polishing, and the application of various protective coatings. Their research showed that these techniques can significantly reduce the surface roughness, but that each has its limitations. Chemical polishing can remove microscopic surface defects, and too much of it can lead to shape deformation. Mechanical grinding, on the other hand, can improve the surface finish, but is time-consuming and can destroy delicate details. Dizon et al. [17] draw attention to the practical aspects of post-processing in 3D printing, analyzing how different post-processing techniques affect the surface roughness and mechanical properties of polymer materials. The article discusses a wide range of post-processing methods used in SLA, emphasizing that obtaining a low-roughness surface is crucial for ensuring performance in industrial applications. The authors state that the appropriate selection of post-processing parameters, such as UV curing, is essential for obtaining a surface with the right smoothness and mechanical resistance. Luna et al. [18] studied the influence of temperature on different aspects of SLA-manufactured elements. During the experiment, the tested objects were manufactured at three different room temperatures. Additionally, elements were divided into two groups, one that after the manufacturing process was rinsed with isopropyl alcohol and another that was additionally post-cured for 10 min with an ultraviolet light-emitting diode. The results obtained from experiments indicate that the surface roughness increases with the higher temperatures of the room in which the SLA printer operates, as well as with applying post-curing. In [19], Katheng et al. described studies on the influence of isopropyl alcohol (IPA) post rinsing on different characteristics of SLA-printed elements, among them on surface roughness. The author used three rinsing methods in the experiments: hand washing and automatic and ultrasonic methods. In each method, three different washing times were utilized, equal to 5, 10, and 15 min. The observed Ra and Sa values tend to increase with longer post-rinsing times, regardless of the method used. This conclusion was additionally confirmed by the testing specimens’ surface morphology using a SEM. Different post-rinsing times and their influence on the roughness of SLA-printed surfaces were tested and described in [20]. Several solutions were chosen for experiments including IPA, Yellow Magic7, ethanol, or Easy Cleaner 3d. For each method, the Ra and Rv parameters were measured, and non-statistically important results were observed.

As can be seen, the presented state-of-the-art surface roughness of SLA-manufactured elements depends on a number of factors. Observations presented in [19] and [20] are unambiguous, so the influence of rinsing times on the surface texture is a subject for further research. In this article, research on the surface roughness of 3D-printed components manufactured using SLA is presented, with a focus on the influence of the duration of rinsing in IPA on the surface quality. The study investigates three distinct photopolymeric materials offered by Formlabs—Gray resin, Tough 2000, and Rigid 10K—each characterized by different mechanical behaviors and typical use cases. The components were printed using the Formlabs Form 3B+ system, designed for high-resolution prototyping and functional parts. Several surface roughness parameters were evaluated, including Ra, Rz, Rt, and RSm, in accordance with the ISO 4287 [21] standards, to assess the impact of the IPA rinsing time on the resulting surface texture.

The subsequent sections of the article are organized as follows. First, the methodology of the experimental procedure is described in detail. Next the selected materials are introduced and the measurement methodology is discussed. Subsequently, the article presents the results of the experimental investigations. The roughness parameters are analyzed for each material and rinsing condition. Finally, the conclusions drawn from the study highlight that the rinsing time in IPA can have a measurable effect on the surface roughness of SLA-printed components.

## 2. Materials and Methods

The methodology for this study involved analyzing the impact of rinsing time in IPA on the surface roughness of 3D-printed samples produced using a Formlabs Form 3B+ printer (Somerville, MA, USA). The utilized device operates on Low Force Stereolithography (LFS) technology. This advanced printing method minimizes the peel forces during layer separation, resulting in improved surface quality and dimensional accuracy compared to conventional SLA systems. The Form 3B+ utilizes a laser system with a wavelength of 405 nm and a spot size of 85 microns, allowing for high-resolution prints with layer thicknesses ranging from 25 to 100 microns.

The investigation utilized three different materials provided by Formlabs (Somerville, MA, USA): Gray, Tough 2000, and Rigid 10K. The chosen resins represent three distinct classes of materials with different mechanical properties, structural compositions, and target applications. The differences between the tested materials based on the manufacturer’s description are summarized in Table 1.

**Figure 1:**
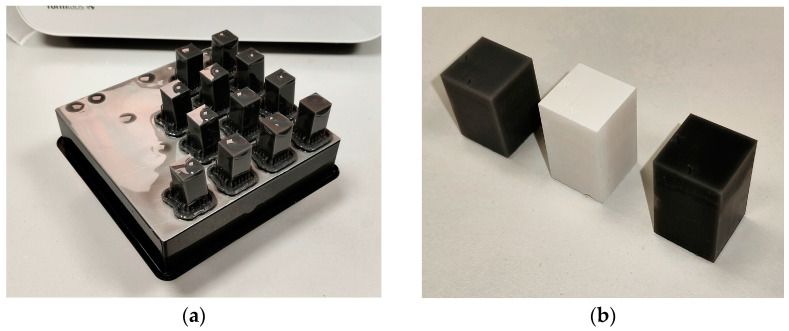
Elements used in described studies: (**a**) parts after manufacturing process; (**b**) parts prepared for roughness measurements. From left to right, specimens made from Gray, Rigid 10K, and Tough 2000 resins.

The geometry of the test objects consisted of a flat rectangular surface area optimized for contact stylus profilometry, ensuring sufficient measurement length and accessibility for multiple profiling passes. The models were exported in the STL format and prepared for printing using the PreForm software 3.46.0, which enabled standardized orientation, support generation, and slicing. Each sample was printed in a vertical orientation, ensuring that the analyzed surface was either perpendicular or inclined (45°) relative to the build platform. Figure 1a presents the specimens right after the printing process, while Figure 1b presents the printed elements after post-curing processes.

**Figure 2:**
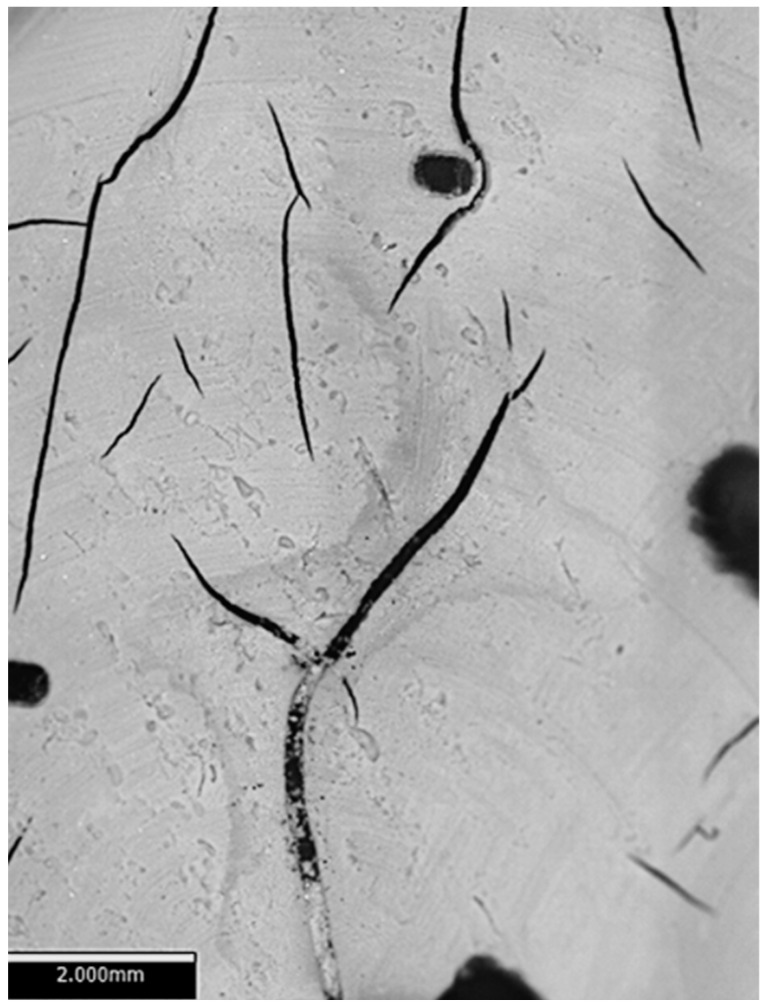
Example of surface cracks caused by inappropriate washing times observed with a Bruker Alicona Infinite Focus G5+.

Next, the parts were post-processed using the Form Wash system filled with analytical-grade IPA at room temperature. Six samples per material were rinsed for varying durations ranging from 6 to 30 min, depending on the resin type. The IPA rinse time was varied to evaluate its influence on the surface roughness. For the Gray material, the rinsing times were set at 6, 8, 10, 12, 15, and 20 min, while for Tough 2000 and Rigid 10K, the rinse times were set as 10, 12, 15, 20, 25, and 30 min. As can be seen for Gray resin, different washing times are applied, because it was observed that for wash times over 20 min, the cracks start to appear on the surfaces of the printed elements. An example of a cracked surface observed with a Bruker Alicona (Raaba/Graz, Austria) focus variation microscope is presented in Figure 2.

Following rinsing, the parts were fully post-cured in the Form Cure system under controlled thermal and UV light exposure: 60 min at 60 °C.

The surface roughness analysis in this study was conducted utilizing the Taylor Hobson Form Talysurf i-Series profilometer (Leicester, UK). The instrument is housed in a specialized room with controlled environmental conditions, which maintains the temperature at a stable 20 °C ± 0.5 °C. The instrument is placed within a custom-built enclosure, constructed from aluminum profiles and Plexiglas panels to isolate the instrument from external environmental disturbances. Furthermore, the profilometer is mounted on a vibration-dampening system and foundation, which isolates it from the rest of the building to further reduce the impact of ambient vibrations.

**Figure 3:**
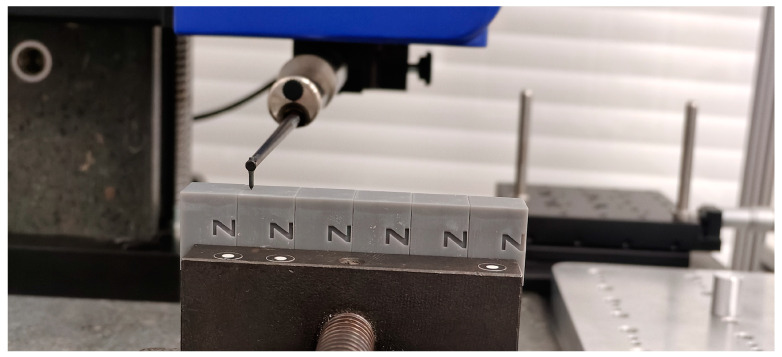
Measurement of surface roughness for specimens made from Gray resin. All six elements are mounted in a vise in order to automate measurement process.

The measurement process involved the measurement of each sample over a 4 mm evaluation length, in accordance with [22]. Five parallel traces were performed on each sample and spaced 0.5 mm apart, to ensure comprehensive surface coverage and to account for any local variations in roughness. The profiles were measured in the direction perpendicular to the build orientation. The same automatic program was applied to all samples to minimize the influence of the measurement procedure on the obtained results. Four roughness parameters were assessed during the experiment: the Ra (arithmetical mean roughness), Rz (maximum height of the profile), Rt (total height of the profile), and RSm (mean spacing between profile irregularities). Figure 3 shows how the samples were assembled in measuring the volume of the utilized device.

## 3. Results

**Figure 4:**
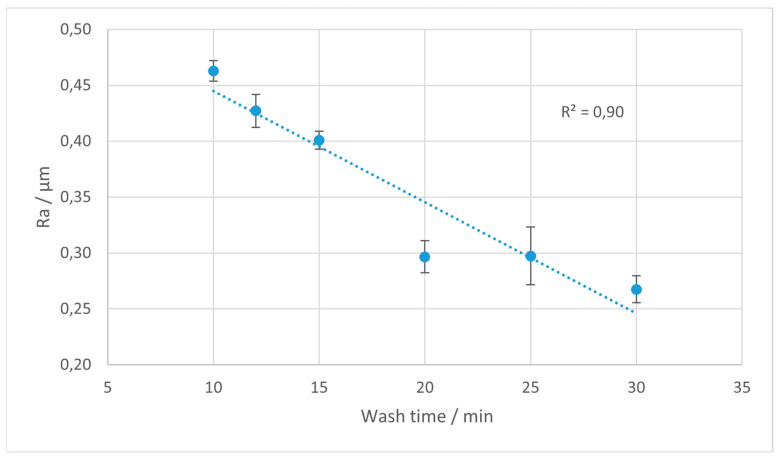
Average values of Ra parameter for Rigid 10K resin depending on the rinsing time. Error bars indicate standard deviations for measurements on each sample. The trend line is marked with the dotted line.

**Figure 5:**
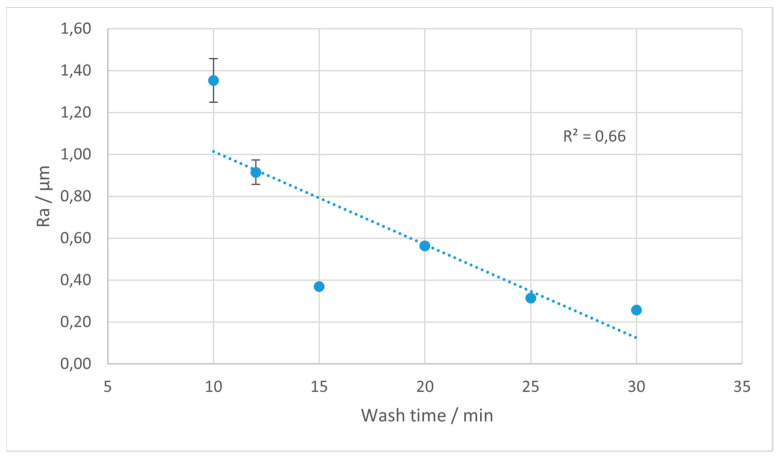
Average values of Ra parameter for Tough 2000 resin depending on the rinsing time. Error bars indicate standard deviations for measurements on each sample. The trend line is marked with the dotted line.

**Figure 6:**
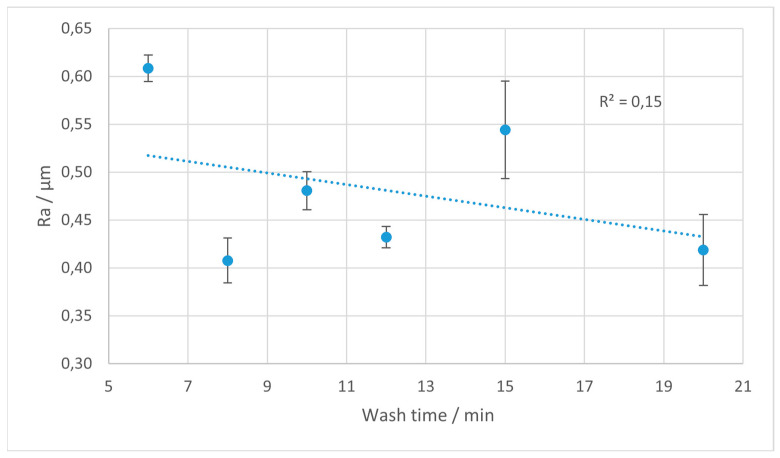
Average values of Ra parameter for Gray resin depending on the rinsing time. Error bars indicate standard deviations for measurements on each sample. The trend line is marked with the dotted line.

**Figure 7:**
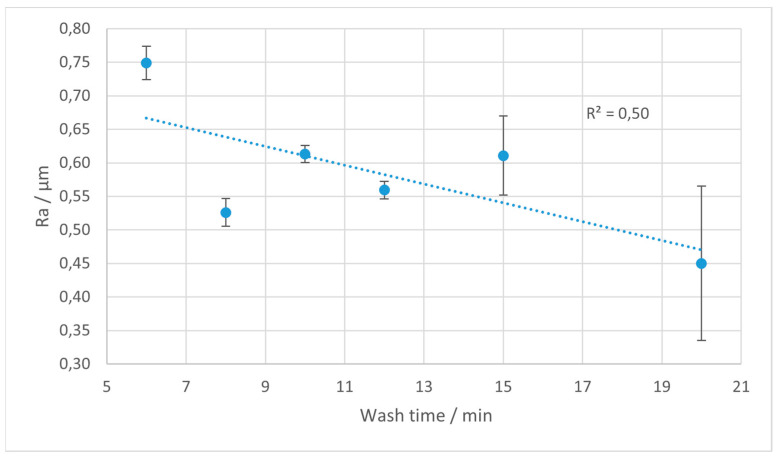
Average values of Ra parameter for Gray 45 resin depending on the rinsing time. Error bars indicate standard deviations for measurements on each sample. The trend line is marked with the dotted line.

The Ra parameter is most often utilized in studies on surface roughness, and is extensively used in industrial practice. In Figure 4, Figure 5, Figure 6 and Figure 7, the results of the Ra parameter measurements are presented. The dots on the graphs represent the average value of Ra obtained from five sections and the error bars are equal to the obtained values of the standard deviations.

For the Ra parameter, which represents the arithmetic average of the absolute deviations from the mean line, a decreasing trend with an increased washing time is observed for the Rigid 10K and Tough 2000 materials. This trend is particularly visible for Rigid 10K, where the Ra values drop markedly with prolonged IPA exposure, reflecting a progressive smoothing effect on the surface profile. The slope of this reduction is most pronounced between 10 and 25 min, after which the values tend to stabilize.

In the case of Tough 2000, a similar overall trend is present, although with greater variability. The Ra values initially show a decline with an increased washing duration, but this reduction is less linear, and at extended rinse times, some measurements show a resurgence of roughness.

For Gray resin, the decrease in the Ra is more moderate and consistent for the vertically printed samples, with changes becoming marginal after 12 min of rinsing. In contrast, Gray printed at 45° shows a more noticeable decline in the Ra with time, especially between 6 and 15 min, after which the characteristic levels out.

In terms of spread (standard deviation), Rigid 10K and Tough 2000 demonstrate low variance, indicating the high consistency of the surface quality regardless of time. Conversely, Gray shows significant dispersion.

All measured parameters are summarized in Table 2, Table 3, Table 4 and Table 5 presented below.

Considering the Rz and Rt, which capture the peak-to-valley characteristics of the profile, the observed trends generally mirror those seen with the Ra. For Rigid 10K, both the Rz and Rt decrease between 10 and 20 min, indicating that extended rinsing reduces both the average and extreme profile deviations.

For Tough 2000, the values of Rz decrease between 10 and 15 min, and for Gray resin, both the Rz and Rt decrease slightly with time, but the rate of change flattens considerably after 12 min. The values remain relatively low throughout, reaffirming the material’s dimensional stability and surface resilience. For specimens built with a 45° orientation, the decline in the Rz is more evident up to 15 min, while the Rt shows little to no significant variation beyond the initial washing phase.

Finally, for the RSm parameter, which describes the mean spacing between the roughness profile elements, the trends vary more significantly by material. Rigid 10K and Tough 2000 exhibit decreasing trends. For Gray and Gray (45°) samples, the RSm remains relatively stable, though the angled prints demonstrate a slight decrease.

In summary, the overall trends indicate that an increased IPA rinsing time generally leads to improved surface quality, particularly for Rigid 10K and Tough 2000. Parameters such as the Ra and Rz respond more predictably to rinsing time, whereas the Rt and RSm act less predictably.

## 4. Discussion

The results suggest that the isopropyl alcohol (IPA) washing time has a significant impact on the surface roughness of parts produced using the SLA method. With increasing washing time, a general decreasing trend was observed for all the analyzed roughness parameters (Ra, Rz, Rt, and RSm), although the extent and nature of these changes depended on the type of material used.

For Rigid 10K, a stable, linear decrease in roughness was observed as the washing time increased, indicating the effective removal of resin residues without the risk of surface degradation. Tough 2000 exhibited the highest initial roughness, but also the greatest potential for improvement—after 15 min, the Ra was reduced by over 70%, and further washing continued to improve the results, although a temporary deterioration was noted at 20 min. Gray resin, as a standard material, reached its lowest Ra values after 8–12 min of washing; the further extension of the process did not yield significant improvements, and a slight degradation was observed at 15 min. Gray samples printed at a 45° angle were consistently rougher than their vertically printed counterparts, which can be attributed to layer geometry and the presence of support structures.

The parameters Rt and RSm confirm these observations. Extended washing reduced the maximum height differences within the surface profile. In cases of temporary deterioration (e.g., Tough 20 min, Gray 15 min), these parameters most likely indicated the appearance of local surface defects, which were resolved after additional rinsing.

The mechanism of surface improvement is based on the removal of uncured resin remaining on the part’s surface. However, excessive washing time may lead to secondary effects, such as polymer swelling, the deposition of dissolved oligomers, or micro-deformations. Therefore, it is necessary to define the optimal washing time, which, based on the results, can be estimated as follows: Gray: 8–12 min, Tough 2000: 15–25 min, and Rigid 10K: 20–30 min.

These conclusions have significant practical relevance—a properly selected washing time enables the achievement of a surface quality comparable to that of mechanically finished parts. In industrial and medical applications, where dimensional accuracy and surface quality are critical, optimizing the washing process may reduce the need for further post-processing and improve the production reliability.

**Figure 8:**
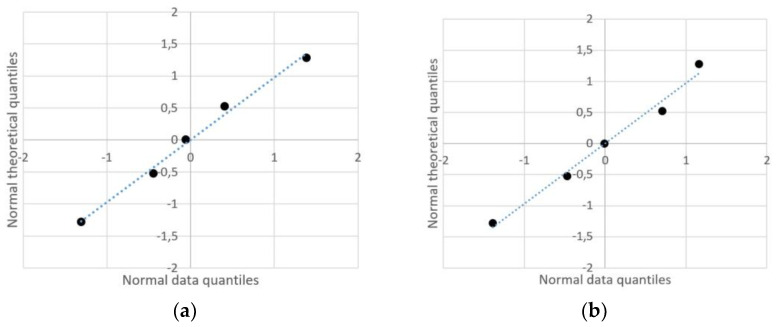
Quantile–quantile plot for two chosen sets of data obtained during experiments: (**a**) Rz roughness parameters obtained for 12 min IPA rinsing for Tough 2000 resin; (**b**) RSm roughness parameters obtained for 15 min IPA rinsing for Gray resin.

In order to determine whether the differences in the tested surface roughness parameters obtained for various IPA washing times were statistically significant, the ANOVA method was applied separately for each material and each parameter. Analysis started with checking the normality distribution of the obtained results using the Shapiro–Wilk test. The obtained results do not provide grounds to reject the hypothesis of normal distribution for any of the tested groups. Figure 8 presents the qq plots obtained for two groups taken as examples.

The ANOVA was conducted using a significance level of 0.05. Table 6 presents the *p*-values for all analyses performed and indicates whether the results can be considered statistically significant.

By analyzing the presented results, it can be stated that in most of the examined cases, the differences between groups can be considered statistically significant. Only in the case of the Rt and RSm parameters for Rigid 10K, as well as for the samples made from Gray resin and printed at an angle, did the analysis not reveal statistically significant differences between the groups. This observation may be attributed to the presence of individual outliers within the analyzed datasets, which likely increased the within-group variability. As a result, despite apparent trends in the data, the ANOVA test did not indicate statistically significant differences between the groups.

Subsequently, a more detailed analysis was conducted using the Tukey HSD test. The critical Q value (Q_crit_) for this test was 4.3729, based on the assumed significance level of 0.05. For the Rigid 10K material, statistically significant differences were observed for nearly all pairwise group comparisons for the Ra parameter, except for the final two washing durations (25 and 30 min), suggesting that extending the washing time beyond this threshold does not further improve the Ra value. A similar trend was observed for the Rz parameter. For the Rt and RSm, the test did not indicate any significant differences between the groups.

In the case of the Tough 2000 material, statistically significant differences were found for nearly all surface roughness parameters across the groups, although the significance tended to diminish between the last two washing durations.

For the Gray resin, in most cases, no statistically significant differences were observed between the groups, regardless of whether the samples were printed vertically or at an angle.

Another possible way to assess the influence of the IPA washing times on the values of the parameters describing the surface roughness is through the determination of the Pearson’s correlation coefficients for each tested parameter. Rigid 10K shows very strong negative correlations for the following parameters: Ra (r = −0.95) and Rz (r = −0.92), indicating that prolonged IPA washing times significantly reduce the surface roughness, as the *p*-values were smaller than 0.01. For the Rt and RSm, no strong correlations were obtained. Tough 2000 also shows negative correlations for the Ra (r = −0.81) and Rz (r = −0.83), and for the Rt and RSm, *p*-values bigger than 0.05 were obtained. At the same time, some data points suggest the risk of surface degradation, such as swelling or micro-cracks, especially under prolonged IPA exposure. This suggests that, in certain cases, extended washing may not always lead to improvements. For Gray resin, samples printed vertically show medium correlations with the Ra (r = −0.38) and Rz (r = −0.42), with *p*-values bigger than 0.05. The correlations for the Rt (r ≈ 0.03) and RSm (r ≈ 0.06) are practically negligible, suggesting that in the case of standard vertical printing, the IPA washing duration does not significantly influence the surface texture. Also, for Gray resin printed at a 45° angle, the calculated correlations coefficients are for Ra (r = −0.71), Rz (r = −0.43) and Rt (r = −0.49) with *p*-values larger than 0.05, suggesting that the layer orientation does not significantly affect the extreme height values of the surface profile.

**Figure 9:**
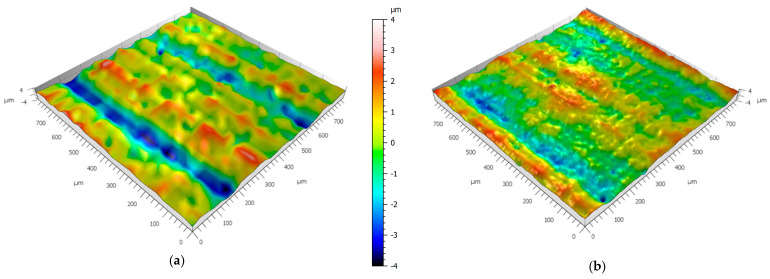
Three-dimensional surface texture obtained from Bruker Alicona for specimens made from Gray resin: (**a**) 6 min washing time and (**b**) 20 min washing time. Scale bar applies to both figures.

**Figure 10:**
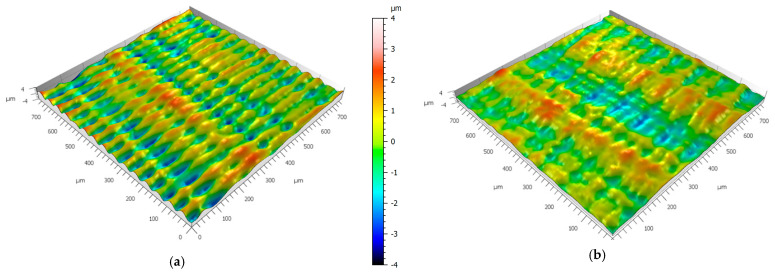
Three-dimensional surface texture obtained from Bruker Alicona for specimens made from Tough resin: (**a**) 10 min washing time and (**b**) 30 min washing time. Scale bar applies to both figures.

The effect of surface smoothing as a function of IPA washing time was further investigated using a Bruker Alicona optical profilometer. To complement the primary roughness measurements, additional analyses were performed on selected samples representing the most pronounced smoothing behavior (Rigid 10K), as well as on samples made from Gray resin, for which the influence of washing time appeared less substantial. For both materials, extreme cases of short and long washing durations were compared. The results were visualized using dedicated three-dimensional topography maps, as presented in Figure 9 and Figure 10.

As can be observed, both materials exhibit clear surface smoothing, manifested by a reduction in the peak heights and valley depths across the scanned surface area. This confirms the quantitative findings from profilometric analysis and provides additional visual evidence of the impact of the post-processing duration on the surface morphology of SLA-printed parts.

## 5. Conclusions

The discussion above emphasizes that the IPA washing time is a controllable factor that markedly affects the surface roughness of SLA prints. Longer wash times generally reduce roughness by removing uncured resin and smoothing the surface, up to an optimal duration specific to each material, beyond which adverse effects may appear. Gray, Tough 2000, and Rigid 10K resins all showed improved Ra, Rz, and Rt values with extended washing, although Tough and Gray exhibited slight mid-course roughness increases, likely due to solvent interactions. The results presented in the article, compared to studies described in the literature, show that the influence of rinsing time on the surface texture should be studied for each material separately, as the findings described, especially in [19], showed different tendencies to results presented in this article. In the cited article, the researchers describe the effect of the rinsing time in IPA on the surface roughness, testing elements made from Clear resin, which is a styrene-acrylic resin, finding an increase in the surface roughness with an extended washing time. When comparing the results obtained in [19] to the studies presented in this article, the polymer with a similar type is the Gray resin. The results for this material show a slight downward trend of Ra values with increasing washing time, but the differences between the results for different washing times are not statistically significant. It should be noted that in [19], the maximum difference between the obtained Ra values was 0.15 μm, while for the Gray resin, it was about 0.2 μm. Considering both of these observations, it can be concluded that for polymers of this type, the rinsing time does not significantly affect the obtained Ra values. It should be noted that although the Clear and Gray resins are of a similar type, they are different materials, and the difference in the auxiliary matters used in them may cause variations in how they react to IPA washing. For other resins presented in this article, the statistical analysis indicates that longer washing times may significantly reduce the values of selected roughness parameters. Isopropyl alcohol acts as a solvent, effectively removing excess uncured material. However, different resins have different chemical sensitivities to this solvent. In the case of resins that are more sensitive to solvents, the alcohol can dissolve or soften the surface of the printed element, causing changes in roughness, such as a smoothing effect.

The studies presented in this paper showed that there are some differences between the trends in the Ra and Rz parameters and Rt parameter, which is determined for longer distances that the first two mentioned. As a direction for future studies that should be undertaken with the highest priority, we identified the analyses of changes in the values of the roughness parameters that should be determined for distances longer than the sampling distance. This will contribute to a better understanding of the changes in the materials used in SLA processes caused by varying the IPA rinsing time.

## Figures and Tables

**Table 1:** Comparison of properties of materials used in experiments.

Property	Rigid 10K	Tough 2000	Gray
Primary Use	High-detail prototyping, visual presentation	Functional prototypes requiring durability and stiffness	High-precision, load-bearing parts with minimal deflection
Surface Finish	Smooth, matte	Slightly textured, durable	Smooth, high detail
Tensile Strength	~62 MPa	~46 MPa	>65 MPa
Modulus of Elasticity	~2.6 GPa	~2.2 GPa	~10 GPa
Elongation at Break	~13%	~48%	~1%
Mechanical Behavior	Brittle, high stiffness	Tough and semi-rigid (ABS-like behavior)	Very stiff, low flexibility

**Table 2:** Mean Ra values (obtained from five measured profiles) obtained for different washing times and for different materials. If not stated otherwise, values are given in µm. SD stands for standard deviation.

Washing Time/min		Rigid 10K		Tough 2000		Gray		Gray Angled	
Rigid 10K or Tough 2000	Gray or Gray Angled								
		Mean	SD	Mean	SD	Mean	SD	Mean	SD
10	6	0.463	0.018	1.352	0.209	0.609	0.028	0.749	0.050
12	8	0.427	0.030	0.915	0.117	0.408	0.047	0.526	0.041
15	10	0.401	0.016	0.370	0.011	0.481	0.040	0.613	0.026
20	12	0.297	0.029	0.564	0.026	0.432	0.023	0.559	0.026
25	15	0.297	0.052	0.314	0.024	0.544	0.102	0.611	0.118
30	20	0.267	0.024	0.257	0.020	0.419	0.074	0.450	0.230

**Table 3:** Mean Rz values (obtained from five measured profiles) obtained for different washing times and for different materials. If not stated otherwise, values are given in µm. SD stands for standard deviation.

Washing Time/min		Rigid 10K		Tough 2000		Gray		Gray Angled	
Rigid 10K or Tough 2000	Gray or Gray Angled								
		Mean	SD	Mean	SD	Mean	SD	Mean	SD
10	6	3.384	0.114	5.555	1.046	3.895	0.143	4.199	0.215
12	8	3.139	0.206	4.862	0.502	2.783	0.303	3.333	0.228
15	10	3.037	0.140	2.283	0.100	3.289	0.173	3.594	0.164
20	12	1.976	0.219	3.653	0.185	2.913	0.222	3.575	0.491
25	15	2.263	0.514	2.085	0.235	3.681	0.547	3.670	0.443
30	20	1.834	0.182	1.647	0.130	2.693	0.326	3.473	0.198

**Table 4:** Mean Rt values (obtained from five measured profiles) obtained for different washing times and for different materials. If not stated otherwise, values are given in µm. SD stands for standard deviation.

Washing Time/min		Rigid 10K		Tough 2000		Gray		Gray Angled	
Rigid 10K or Tough 2000	Gray or Gray Angled								
		Mean	SD	Mean	SD	Mean	SD	Mean	SD
10	6	6.753	1.464	7.285	1.499	6.499	0.751	7.039	0.685
12	8	5.578	0.884	10.666	1.182	5.145	0.955	6.510	1.160
15	10	6.034	1.772	3.783	0.526	5.798	1.007	6.424	0.797
20	12	3.386	0.966	6.897	0.965	6.023	1.815	8.653	1.414
25	15	5.716	1.221	3.990	1.029	10.071	1.758	9.373	0.906
30	20	2.976	0.177	3.391	1.126	4.498	0.440	6.096	0.510

**Table 5:** Mean RSm values (obtained from five measured profiles) obtained for different washing times and for different materials. If not stated otherwise, values are given in mm. SD stands for standard deviation.

Washing Time/min		Rigid 10K			Tough 2000				Gray			Gray Angled	
Rigid 10K or Tough 2000	Gray or Gray Angled												
		Mean		SD	Mean			SD	Mean		SD	Mean	SD
10	6	0.024		0.002	0.054			0.008	0.033		0.003	0.050	0.006
12	8	0.022	0.002		0.057		0.003		0.035	0.003		0.040	0.002
15	10	0.027	0.003		0.043	0.003			0.034	0.003		0.041	0.003
20	12	0.021	0.002		0.040	0.003			0.032	0.004		0.047	0.012
25	15	0.024	0.009		0.039	0.004			0.042	0.007		0.044	0.010
30	20	0.019	0.001		0.043	0.006			0.031	0.004		0.039	0.003

**Table 6:** The *p*-values obtained from ANOVA of each tested material and roughness parameters. Green color indicates statistically significant differences between tested groups and red color was used for indicating insignificant differences.

**Material**	**Roughness Parameter**			
	Ra	Rz	Rt	RSm
Rigid 10K	0.00	0.00	0.06	0.25
Tough 2000	0.00	0.00	0.00	0.00
Gray	0.01	0.00	0.00	0.01
Gray angled	0.01	0.01	0.44	0.16

## Data Availability

The original contributions presented in this study are included in the article. Further inquiries can be directed to the corresponding author.

## Abbreviations

The following abbreviations are used in this manuscript:

SLA	Stereolithography
AM	Additive manufacturing
SEM	Scanning Electron Microscope
IPA	Isopropyl alcohol
